# Repeated transarterial chemoembolisation using different chemotherapeutic drug combinations followed by MR-guided laser-induced thermotherapy in patients with liver metastases of colorectal carcinoma

**DOI:** 10.1038/bjc.2012.69

**Published:** 2012-03-01

**Authors:** T J Vogl, A Jost, N A Nour-Eldin, M G Mack, S Zangos, N N N Naguib

**Affiliations:** 1 Institute for Diagnostic and Interventional Radiology, Johann Wolfgang Goethe-University, Theodor-Stern-Kai 7, 60590 Frankfurt am Main, Germany; 2 Department of Radiology, Cairo University Hospital (Kasr Elainy), Cairo, Egypt; 3 Department of Radiology, Alexandria University Hospital, Alexandria, Egypt

**Keywords:** colorectal cancer liver metastases, chemoembolisation, laser-induced thermotherapy

## Abstract

**Background:**

To evaluate a treatment protocol with repeated transarterial-chemoembolisation (TACE) downsizing before MR-guided laser-induced interstitial thermotherapy (LITT) using different chemotherapeutic combinations in patients with unresectable colorectal cancer (CRC) liver metastases.

**Methods:**

Two hundred and twenty-four patients were included in the current study. Transarterial-chemoembolisation (mean 3.4 sessions per patient) was performed as a downsizing treatment to meet the LITT requirements (number⩽5, diameter <5 cm). The intra-arterial protocol consisted of either Irinotecan and Mitomycin (*n*=77), Gemcitabine and Mitomycin (*n*=49) or Mitomycin alone (*n*=98) in addition to Lipiodol and Embocept in all patients. Post TACE, all patients underwent LITT (mean 2.2 sessions per patient).

**Results:**

Overall, TACE resulted in a mean reduction in diameter of the target lesions of 21.4%. The median time to progression was 8 months, calculated from the start of therapy and the median local tumour control rate was 7.5 months, calculated as of therapy completion. Median survival of patients calculated from the beginning of TACE was 23 months (range 4–110 months), in patients treated with Irinotecan and Mitomycin the median was 22.5 months, Gemcitabine and Mitomycin 23 months and Mitomycin only 24 months with a statistically significant difference between the groups (*P*<0.01).

**Conclusion:**

Repeated TACE offers adequate downsizing of CRC liver metastases to allow further treatment with LITT. The combined treatment illustrates substantial survival rates and high local tumour control with statistically significant differences between the three protocols used. Further randomised trials addressing the current study results are required.

Colorectal cancer (CRC) is the third leading cause of cancer death worldwide with an overall cancer mortality of 610 000 deaths each year (WHO, 2009), with the liver as the most common site of metastases. The hepatic tumour load is an important prognostic indicator for survival because the involvement of the liver is life limiting. Currently, surgical resection is considered to be the only potentially curative therapy, but only 15–20% of the patients are suitable for surgical resection (Fong *et al*, 1999; Golling and Bechstein, 2006) due to advanced disease process or secondary disorders.

Regional treatments are a promising alternative to terminate the growth of the metastases and to extend the survival in patients (Solbiati *et al*, 2001; Christophi *et al*, 2004; Siperstein *et al*, 2007) who are not qualified for surgical resection. Although transarterial chemoembolisation (TACE) is considered a palliative treatment for patients with advanced tumour disease, ablation methods have provided success rates comparable to surgical resection (Christophi *et al*, 2004; Vogl *et al*, 2004; Pech *et al*, 2007). Still, these methods are only applicable under certain conditions, namely size and number of the lesions to be ablated (for laser-induced interstitial thermotherapy (LITT): size <5 cm, number ⩽5) (Vogl *et al*, 2002).

In preceding studies, the use of TACE as a palliative treatment in patients with liver metastases of CRC (Vogl *et al*, 2007) was assessed. Other studies focused on the ablative alternatives for CRC liver metastases (Solbiati *et al*, 2001; Christophi *et al*, 2004; Vogl *et al*, 2004; Pech *et al*, 2007; Siperstein *et al*, 2007). The combination of TACE as a downsizing method followed by LITT has only been investigated in a small patient group with one intra-arterial protocol (Vogl *et al*, 2003). Accomplishing further data in a large cohort particularly concerning CRC liver metastases and comparing three different chemotherapeutic drug combinations is considered necessary.

Based on this, the aim of the current study was to assess the therapeutic value of TACE as a downsizing therapy before LITT using different chemotherapeutic drug combinations in patients with liver metastases of CRC. A focus was set on the survival data, the rate of local intrahepatic tumour progression and the development of *de novo* lesions.

## Patients and methods

### Patients

Between January 1999 and September 2008, 224 patients (143 male (63.8%) and 81 female (36.2%)) in the mean age of 61.2 years (range 35–87 years, s.d.: 9.87) with liver metastases of colorectal carcinoma underwent 757 TACE sessions (mean 3.4 sessions per patient, s.d. 1.33, range 1–10). All patients included in this study were treated with LITT for post-TACE remaining lesions (total 492, mean 2.2 sessions per patient, s.d. 1.35, range 1–7).

### Study design and eligibility criteria

The study was approved by the Ethical Committee and informed consent was obtained from all patients included.

Included were patients with liver metastases of CRC origin, which are recurrent after partial liver resection, locally nonresectable or in both liver lobes and deemed by the gastroenterology surgeons to be inoperable. The inclusion criteria also comprised patients with existing contraindications for surgery or refusal of surgical treatment or those who showed adverse reactions or no response to systemic chemotherapy (defined as disease progression under systemic chemotherapy according to the RECIST criteria). Exclusion criteria for the combined TACE and LITT protocol were the presence of extrahepatic metastases, tumour load more than 70% of the liver, poor general condition (Karnofsky <70%), poor hepatic synthesis (serum albumin level <2 mg dl^−1^), high serum bilirubin level (>3 mg dl^−1^), renal insufficiency (serum creatinine level >2 mg dl^−1^), presence of ascites, partial or complete thrombosis of the main portal vein and respiratory or cardiovascular failure. To be eligible for LITT, patients were required to have five or less lesions, none larger than 5 cm in diameter.

### Imaging technique

All imaging studies were performed using a 1.5 Tesla MR System (Magnetom Symphony or Avanto; Siemens, Erlangen, Germany). For initial tumour planning, non-enhanced and contrast-enhanced MR imaging with 0.1 mM kg^−1^ body weight of Gd-DTPA (Magnevist, Schering, Berlin, Germany) was performed in all patients. The MR-imaging protocol included T1-weighted gradient-echo sequences (FLASH-2D) with transverse and sagittal slice orientation (TR/TE: 135/6 ms; FA 80° FOV 350 mm; matrix 134 × 256; slice thickness 8 mm; interslice gap 0.8 mm) before or after each intervention. In addition, non-enhanced T2-weighted breath-hold turbo-spin-echo sequences (TR/TE: 3800/92 ms; FA 150° FOV 350 mm; matrix 115 × 256; slice thickness 8 mm; interslice gap 0.8 mm) and contrast-enhanced dynamic VIBE sequences (TR/TE: 4.5/1.8 ms; FA 15° FOV 350 mm; matrix 128 × 256; slice thickness 8 mm; interslice gap 0.8 mm) were performed for the differentiation of the lesions. Post TACE, non-enhanced CT examinations using a four-slice CT scanner (Somatom plus 4, Siemens) with 8 mm slice thickness were performed.

### Transarterial-chemoembolisation treatment

All chemoembolisation procedures were done by one interventionist, with more than 10 years experience in interventional radiology at the beginning of the study. The procedures were carried out using an Axiom Multistar or Axiom Artis system (Siemens).

After the introduction of a selective catheter through the femoral artery using the Seldinger technique, vascular anatomy of the hepatic arteries was determined. Then, the catheter was advanced beyond the gastroduodenal artery. Depending on size, location and arterial supply of the lesion, the tip of the catheter was advanced further into the segmental or subsegmental arteries. No patient underwent global TACE. Segmental or subsegmental chemoembolisation was performed in all patients.

### Treatment protocol and rational for group assignment

The chemotherapeutic suspension consisted of one of the following chemotherapeutic drug combinations. Group 1 (98 patients) received a suspension of 8 mg m^−2^ b.s. Mitomycin C alone, in group 2 (49 patients) the suspension consisted of a combination of 8 mg m^−2^ b.s. Mitomycin C and 1000 mg m^−2^ b.s. Gemcitabine and group 3 (77 patients) received a combination of 8 mg m^−2^ b.s. Mitomycin C and 150 mg m^−2^ b.s. Irinotecan.

The selection criteria for the chemotherapeutic protocol were as follows: Patients who had undergone systemic chemotherapy protocols containing both Oxaliplatin and Irinotecan (FOLFOX and FOLFIRI, respectively) were treated with Mitomycin alone (*n*=98). Patients who had undergone a previous protocol containing Irinotecan were treated with a combination of Mitomycin and Gemcitabine (*n*=49) and patients who had undergone Oxaliplatin-based treatment were treated with a combination of Mitomycin and Irinotecan (*n*=77). The chemotherapeutic choice was based on a multidisciplinary tumour board including radiologists, oncologists and surgeons.

In all patients, vascular occlusion using 200–450 mg starch microspheres (EmboCept, Pharmacept, Berlin, Germany) was performed after the injection of the chemotherapeutic drug suspension and iodoised oil (Lipiodol, Guerbet, Sulzbach, Germany) (maximum dose of 10 ml), under fluoroscopic control until stasis was observed. After embolisation, the devascularisation of the tumour was confirmed via angiography. Lipiodol retention in the tumour was verified within the first 6 hours after chemoembolisation with nonenhanced CT examinations. Further follow-up examinations were performed with non-contrast-enhanced MRI directly before each TACE session.

Patients who did not meet the LITT requirements (*n*=191) at the beginning were repeatedly treated with at least three sessions (range 3–10, mean 3.7 sessions per patient) in 4-week intervals until they met the above-defined LITT criteria. Patients who were eligible for LITT from the beginning (*n*=33) received 1–2 sessions of TACE (mean 1.6 per patient) to maximise the ablative effect of LITT (Whelan *et al*, 1995; Maataoui *et al*, 2005).

### Laser-induced interstitial thermotherapy treatment

All patients included in this study underwent MR-guided LITT 4 weeks after the final TACE session. All laser therapy procedures were done by two radiologists, with more than 8 years experience at the beginning of the study.

The puncture was performed with CT guidance under local anaesthesia plus an intravenously injected hypnoanalgesic (pethidine). The application set (Somatex, Berlin, Germany) consisted of a cannulation needle with a tetragonally sharpened tip, a guide wire, a double-laminated 9-F-sheath-catheter (length 20 cm) with mandrin and a special protective 9-F-catheter (length 43 cm), which was closed at its distal end. The protective catheter had a double lumen with the flexible fibre that delivered the laser light in the centre and saline (at room temperature) circulating around the fibre to constantly cool it. This system prevented direct contact of the laser applicator with the patient to increase the safety. The catheter was temperature-resistant and permeable to laser light. For the thermal ablation, patients were immediately transported to the adjoining MR room because LITT was performed under MR guidance. A Nd:YAG laser with 1064 nm wave length, which operated in continuous wave mode was used. The laser energy varied from 20 to 35 W and the period of application ranged from 10 to 35 min. The laser applicators were positioned with respect to size and location of the metastasis. For large lesions, multiple applicators were used. During the treatment, the temperature development and distribution was monitored by a thermo-sensitive T1-weighted FLASH-2D sequence (TR/TE: 140/12 ms; FA 80° FOV 350 mm; matrix 128 × 200; slice thickness 8 mm; interslice gap 0.8 mm; acquisition time 15 s) to ensure complete necrosis of the lesion seen as signal loss. Immediately after the coagulation, contrast-enhanced FLASH-2D sequences (TR/TE: 140/12 ms; FA 80° FOV 350 mm; matrix 128 × 200; slice thickness 8 mm; interslice gap 0.8 mm) were performed to document the laser-induced necrosis and possible complications. Following the intervention, the puncture channel was blocked by fibrin glue (Tissucol Duo S 2 ml, ImmunoBaxter, Unterschleissheim, Germany).

After the treatment, the patients were monitored for 6 hours in case any possible adverse effects occurred. The observation consisted of a monitoring of the vital parameters and a clinical examination. They were discharged on the same day of ablation if no complications were noted. Within the following 24 h, a control MRI was done. Further follow-up MRI examinations were performed in 3-month intervals. Patients were followed-up for a mean of 10.74 months (range 1–73 months, s.d. 10.94).

### Data collection

All responses were based on findings at MR imaging. All measurements were calculated by two radiologists (with more than 5 and 10 years of experience in abdominal imaging) in consensus. The change in size was calculated by radiological evaluation using MRI and the response was evaluated according to the RECIST system (Eisenhauer *et al*, 2009) based on the longest dimension of the target lesions. The clinical data were recorded as provided to us by the referring oncologists. The complications and adverse events were documented from the obtained imaging studies and from post-interventional monitoring and were assessed using the NCI Common Terminology Criteria for Adverse Events (CTCAE v3.0). Abdominal pain, fever, nausea and vomiting after TACE were considered as postembolisation syndrome.

The time to progression, the survival rate and the local tumour control rate were recorded as well. The time to progression included the development of *de novo* metastases and local intrahepatic tumour progression, whereas the local tumour control rate only referred to local tumour progression on previously ablated areas. The time to progression was defined as starting from the beginning of TACE therapy and the local tumour control rate was defined as starting as of therapy completion (last LITT session). The ending was either occurrence of progression or local tumour progression or for patients lacking progression or local tumour progression, the last follow-up examination available at the time of evaluation. The survival rate was defined as starting as of the first TACE treatment and ending when death of the patient occurred, or for living patients at the last follow-up examination available at the time of evaluation.

### Quantitative and statistical analysis

Survival rates were calculated using the Kaplan–Meier method. The difference between the three groups concerning survival rates, local tumour control and time to progression was tested with the Log-rank test. The significance concerning reduction in tumour size was calculated with the Wilcoxon-matched-pairs test and the difference in tumour size reduction between the three chemotherapeutic drug groups was evaluated with the Kruskal–Wallis test. *P*-values <0.05 were considered statistically significant.

The response to the treatment was evaluated according to the RECIST system, once before the first LITT treatment to classify the response to the TACE therapy and at the 6-month follow-up (or at the last available follow-up) of the laser therapy to assess the response to the combined therapy. Complete response after LITT treatment was defined as absence of enhancement in the coagulation zones with no new lesions seen. Furthermore, the local tumour progression rate of previously ablated lesions and the development of *de novo* metastases were documented.

## Results

### Findings after TACE

Post-interventional evaluation after TACE showed partial response in 70 patients (31.25%), whereas stable disease was observed in 154 patients (68.75%). In spite of the RECIST criterion stable disease, the metastases showed sufficient reduction in size to be eligible for LITT therapy. The MRI scans before TACE treatment showed a mean diameter of the lesions of 4.07 cm (range 0.3–8.8 cm, s.d.: 1.69). After the final TACE treatment, the mean diameter of the lesions was 3.20 cm (range 0.3–5 cm, s.d.: 1.27). This results in a mean of 21.4% reduction in the diameter. The reduction in size was statistically significant (*P*<0.001).

**Figure 1:**
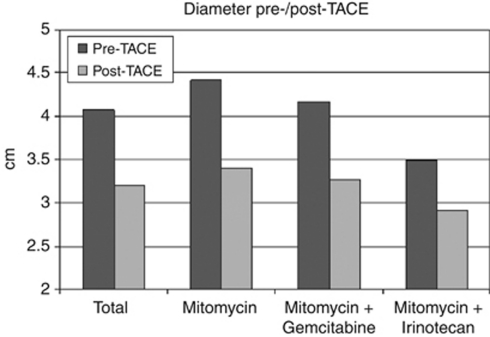
Bar graph showing the mean diameters of the lesions after TACE (light grey) compared with the mean diameters before TACE (dark grey). For each patient group, the obtained data is represented as two columns grouped together. The groups of columns are ordered from left to right as follows: results of all metastases, results of those treated by Mitomycin, results of those treated by Mitomycin-Gemcitabine and results of those treated by Mitomycin-Irinotecan.

In group 1, the mean diameter of the lesions before TACE treatment was 4.42 cm (range 0.3–8.8 cm, s.d.: 1.76). After the final TACE treatment, the mean diameter of the lesions was 3.40 cm (range 0.3–5 cm, s.d.: 1.38), thus showing a mean of 23.1% reduction in the diameter. The reduction in size was statistically significant (*P*<0.001). In group 2, the mean diameter of the lesions before TACE treatment was 4.16 cm (range 2–8 cm, s.d.: 1.54). After the final TACE treatment, the mean diameter of the lesions was 3.27 cm (range 1.2–5 cm, s.d.: 1.16), thus showing a mean of 21.4% reduction in the diameter. The reduction in size was statistically significant (*P*<0.001). In group 3, the mean diameter of the lesions before TACE treatment was 3.49 cm (range 1.3–7.3 cm, s.d.: 1.41). After the final TACE treatment, the mean diameter of the lesions was 2.92 cm (range 1–5 cm, s.d.: 1.17), resulting in a mean of 16.3% reduction in the diameter (Figure 1). The reduction in size was statistically significant (*P*<0.001). No statistically significant difference between the three chemotherapeutic protocols regarding tumour size reduction (tumour diameter) was found (*P*=0.72). Although the sizes of the three groups are not similar, they were comparable in demographic data and mean number of TACE and LITT sessions per patient (Table 1).

### Findings after LITT

Post-interventional MRI studies revealed complete response in 54 patients (24.1%), partial response in 19 patients (8.5%) and stable disease in 16 patients (7.1%). Disease progression after initial stabilisation was found in 135 patients (60.3%), resulting in additional TACE treatments in 107 patients using the same chemotherapeutic drugs used before LITT. The post-TACE remaining lesions were treated with 492 LITT treatments (mean 2.2, s.d. 1.35, range 1–7). In all lesions, complete ablation was observed in the 24-h follow-up MRI after LITT.

### Adverse events

The patients tolerated the TACE procedure well. No fatal or major complications occurred. The side effects after TACE were mild (no or few symptoms), only a small group of patients had symptoms considered as postembolisation syndrome (grade 1/2 adverse events), which were transient and responded to medical treatment. All patients were discharged on the same day of the treatment. After LITT, the rate of clinically relevant complications requiring further interventions was 0.8% (*n*=4). Those complications were conservatively treated in three of the four cases (two pleural effusions treated with an aspiration (grade 2), one bilioma requiring drainage (grade 3)), except for one intraabdominal bleeding that was intraoperatively stilled (grade 3). The most common minor complication (defined as requiring no intervention) of LITT was reactive pleural effusion in 27.4% (*n*=135) that was self-limited. Other minor complications were small bilioma developing consecutive to LITT in 3.7% of the LITT treatments (*n*=18), subcapsular haematoma, which was observed in 4.9%, (*n*=24) and small lung atelectasis, which was observed in 5.7% (*n*=28). None of these required any further treatment. There was no procedure-related mortality. No seeding of metastases along the cannulation tract was found. Table 2 summarises the procedure-related clinical adverse events.

### Local tumour control and time to progression

Time to progression showed a median value of 8 months (interquartile range (IQR) 6–12.25, range 0–80 months) from the beginning of therapy. In group 1, the time to progression showed a median of 8 months (IQR 6–11.75, range 2–80 months), group 2 showed a median value of 8 months (IQR 6–12, range 1–47 months) and in group 3, it showed a median of 9 months (IQR 6–15, range 0–51 months). No statistically significant difference between the groups regarding the time to progression was found (*P*=0.43).

**Figure 2:**
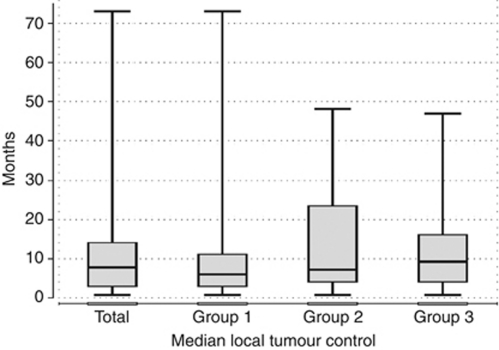
Box plot illustrating the median local tumour control in months with 25 and 75% quartiles calculated from last LITT treatment. The columns are ordered from left to right as follows: results of all metastases, results of those treated by Mitomycin, results of those treated by Mitomycin-Gemcitabine and results of those treated by Mitomycin-Irinotecan.

The overall local tumour control rate, referring only to absence of local tumour progression in previously ablated areas, showed a median of 7.5 months (IQR 3–14, range 0–73 months). In group 1, the overall local tumour control rate showed a median of 6 months (IQR 3–11, range 0–73 months), in group 2, it showed a median value of 7 months (IQR 4–23, range 0–48 months) and in group 3, it showed a median value of 9 months (IQR 4–16, range 0–47 months) (Figure 2). The differences between the three groups regarding the local tumour control were statistically significant (*P*=0.047). During the follow-up, local tumour progression could be observed in 10 of 464 ablated metastases (2%) in 8 patients (3.6%) and newly developed metastases could be found in 133 patients (59.4%), including 6 patients who developed local tumour progression as well, leading to additional TACE treatments in 107 patients.

### Survival analysis

**Figure 3:**
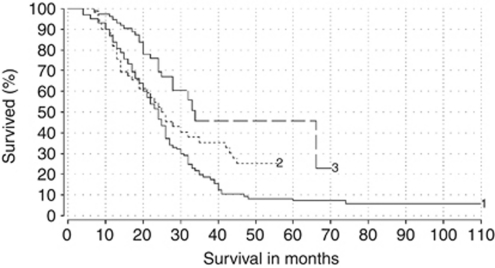
Kaplan–Meier survival curves demonstrating the survival of the patients divided in three groups by virtue of the chemotherapeutic drug used: Group 1 received Mitomycin (*n*=98, median survival 24 months). Group 2 treated with a Mitomycin-Gemcitabine combination (*n*=49, median survival 23 months). Group 3 treated with a Mitomycin-Irinotecan combination (*n*=77, median survival 22.5 months). Log-rank test results showed a statistically significant difference between the three groups of patients (*P*<0.01).

The overall 1-year survival rate was 88%, the 2-year survival rate was 49%, the 3-year survival rate was 19% and the 5-year survival rate was 4.5%. The median survival in months, calculated from the date of the first chemoembolisation treatment, was 23 months (IQR 16–32, range 4–110 months). Comparing the different chemotherapeutic drug combinations used, the median survival of patients in group 1 was 24 months (IQR 15.25–32, range 4–110 months), in group 2, it showed a median value of 23 months (IQR 13.25–42.75, range 7–56 months) and in group 3, it showed a median of 22.5 months (IQR 17–28, range 7–70 months) (Figure 3). The differences regarding the survival were statistically significant (*P*<0.01).

At the end of the evaluation, 67% of all patients (*n*=150) were dead, whereas 33% of the patients (*n*=74) were still alive. In group 1, 92.9% of the patients (*n*=91) died, whereas 7.1% (*n*=7) were still alive, in group 2, 69.4% of the patients (*n*=34) were dead, whereas 30.6% (*n*=15) were still alive and in group 3, 32.5% of the patients (*n*=25) died, whereas 67.5% (*n*=52) are still alive.

## Discussion

As stated before, the liver involvement is life limiting for oncology patients and at the current stage the only curative therapy is surgical resection, which is not possible for around 75% of the patients (Golling and Bechstein, 2006). Additionally, liver surgery is associated with a relatively high morbidity and mortality rate. An operative mortality rate of 1.5% and morbidity rates of 25.9–33.3% (Capussotti *et al*, 2007; Rees *et al*, 2008) were reported. Minimally invasive interventional techniques that can be carried out on an outpatient basis and are associated with fewer complications are a promising therapy option for the above-described patients with liver metastases.

There are two most commonly used local therapeutic approaches for the treatment of the metastases: One is the transarterial administration of chemotherapeutic agents, either as hepatic chemoperfusion or combined with the occlusion of the vascular supply (transarterial chemoembolisation (TACE)). The other local therapeutic approach is the percutaneous access for local tumour destruction, including radiofrequency ablation, cryotherapy, microwave ablation and LITT. Currently, all methods of local ablation are limited by the size of the ablation zones developing after the procedure. To ensure complete ablation without residual tumour tissue, a safety margin of 1 cm to the tumour is necessary. Thus, the maximum size that can be ablated with LITT is 5 cm.

By embolising the hepatic artery, the blood flow is reduced leading to ischaemia, which increases the contact time between the tumour cells and the chemotherapeutic agent (Heisterkamp *et al*, 1997; Albrecht *et al*, 1998; Wacker *et al*, 2001; Ritz *et al*, 2007). In addition, the thermal effect is increased, as the cooling effect of the blood flow, which results in a conservation of viable cells around larger vessels due to a local under heating (Whelan *et al*, 1995), is held off. Hence, TACE combined with MR-guided LITT ablation increases the effectiveness of each of the treatments alone. For these reasons, a small group of patients in our study underwent 1–2 TACE sessions before LITT, although they were suitable for LITT from the beginning.

In metastases that are too large for LITT alone, the downsizing of large liver metastases with TACE is a good method to meet the indications of LITT. Another newly developed option to downsize liver metastases to permit an ablation is the selective internal radioembolisation (Hoffmann *et al*, 2010).

Considering the complications of our treatment protocol in comparison with the results as reported in the major surgical series, it shows that the combination of TACE and LITT, with no treatment-related mortality, a clinically relevant complication rate of 0.8%, grade 3 adverse events in <0.01% and no grade 4 or 5 adverse events, is a safe option to the above-mentioned operative mortality and morbidity rate (Capussotti *et al*, 2007; Rees *et al*, 2008).

Until now, patients with unresectable hepatic metastases of CRC receive either systemic or regional chemotherapy. The median survival rates reported in these patients range between 10 and 20 months (Cassidy *et al*, 2008; Sobrero *et al*, 2008). The mean survival of untreated patients with liver metastases is 4.5–6.6 months (Wood *et al*, 1976; Bengtsson *et al*, 1981).

With TACE offering lesser complications than a systemic chemotherapy and with LITT as end therapy to improve the survival and the local tumour control, our data showed a median survival of 23 months (IQR 16–32, range 4–110 months) from the beginning of the chemoembolisation therapy. The median local tumour control in our study was 7.5 months and the local tumour progression rate was 2%. Laser-induced interstitial thermotherapy offers better local tumour control rates than other minimally invasive techniques (Vogl *et al*, 2004; Pech *et al*, 2007). With radiofrequency ablation, survival rates from 24 to 39 months and local tumour progression rates from 39 to 44% were achieved (Solbiati *et al*, 2001; Abdalla *et al*, 2004; Siperstein *et al*, 2007), with microwave ablation, survival was 27 months and the local tumour progression rates were 15% (Shibata *et al*, 2000; Beppu *et al*, 2001). The comparison of the survival rates does not seem to be feasible because the patients included in this study were in a more advanced stage of the disease than those included in the studies referring to single ablation methods.

Surgical resection in patients with colorectal liver metastases results in a 5-year-survival rate ranging from 29 to 36% (Mutsaerts *et al*, 2005; Capussotti *et al*, 2007; Rees *et al*, 2008) as reported in the major surgical series but patients after hepatectomy need to be hospitalised for a mean duration of 10.4 days (Capussotti *et al*, 2007), whereas TACE and LITT represent therapies that are performed on an outpatient basis. Compared with the 5-year-survival rate in surgical studies, it is well noted that the 5-year-survival rate in this study is much less. However, it is worth mentioning that most of the patients included in this study were not eligible for surgery due to an advanced stage of the disease.

It is worth mentioning that our results should be further evaluated using a randomised trial because the Kaplan–Meier curve (Figure 3) indicates better survival rates for the Mitomycin-Gemcitabine combination and especially for the Mitomycin-Irinotecan combination. This is due to the fact that at the time of evaluation, 31% (15 out of 49) of the patients receiving Mitomycin C-Gemcitabine and 67.5% (52 out of 77) of the patients receiving Mitomycin-Irinotecan were still alive, whereas only 7% (7 out of 98) of the patients treated with Mitomycin C were alive. To understand those differences in survival, it is worth mentioning that in the beginning of this study, the referrals of the oncologists were limited to patients who were highly resistant to multiple systemic chemotherapies and therefore received the Mitomycin-only protocol. Based on the acceptable initial results of the TACE therapy, the referrals were extended to patients who showed only single systemic drug resistance and were consequently eligible for a combined intra-arterial protocol. As a result, it is to be assumed that the survival in the Mitomycin C-Gemcitabine group and especially the survival in the Mitomycin C-Irinotecan group would exceed that of the Mitomycin C-only group if the follow-up was longer.

One limitation to our study is that it is a non-randomised protocol: A randomised study should further evaluate and confirm the promising results of the current study. However, the above-mentioned selection criteria, based on the previous systemic chemotherapies and resistance constitutes a barrier for randomisation. Another limitation is that the tumour response was only assessed in a dimensional assessment according to the RECIST system. In further studies, the functional response should be evaluated via imaging techniques like PET, DWI and so on. Still, the RECIST system is the only internationally accepted response evaluation system.

The large cohort presented in this study confirms that the combination of TACE and MR-guided LITT is a safe and effective treatment for liver metastases of CRC origin.

The combination of TACE and LITT is a good therapy option both for patients not responding to only systemic chemotherapy, and also as an alternative to surgery when the liver resection is contraindicated as it showed no major side effects or complications and is a minimally invasive therapy that can be carried out on an outpatient basis.

## Figures and Tables

**Table 1:** Baseline characteristics of patients

**Characteristic**	**All patients**	**Group 1**	**Group 2**	**Group 3**
*Number of patients*	224	98	49	77
Male	143 (63.8%)	61 (62.2%)	30 (61.2%)	52 (67.5%)
Female	81 (36.2%)	37 (37.8%)	19 (38.8%)	25 (32.5%)
				
*Age*				
Mean	61.2	62.1	59.9	61.0
Range	35–87	35–87	37–86	36–78
				
*Number of TACE*	757	304	174	279
Mean sessions per patient	3.4	3.1	3.5	3.7
				
*Number of LITT*	492	216	107	169
Mean sessions per patient	2.2	2.2	2.14	2.2
				
Previous systemic chemotherapy		Oxaliplatin Irinotecan	Irinotecan	Oxaliplatin

Abbreviations: LITT, laser-induced interstitial thermotherapy; TACE, transarterial-chemoembolisation.

**Table 2:** Procedure-related clinical adverse events

**Adverse event**	**All grades**	**Grade 1**	**Grade 2**	**Grade 3**	**Grade 4**	**Grade 5**
Pleural effusion	137 (27.8%)	135 (27.4%)	2 (*P*<0.01)	0	0	0
Bilioma	19 (3.9%)	18 (3.7%)	0	1 (*P*<0.01)	0	0
Subcapsular hematoma	24 (4.9%)	24 (4.9%)	0	0	0	0
Lung atelectasis	28 (5.7%)	28 (5.7%)	0	0	0	0
Intraabdominal bleeding	1 (*P*<0.01)	0	0	1 (*P*<0.01)	0	0
